# “*My home garden is a peace maker”*: perceived impact pathways of a home gardening intervention in rural Kenya: the ALIMUS study

**DOI:** 10.1186/s41043-026-01263-4

**Published:** 2026-02-15

**Authors:** Erick Agure, Grace Wothaya Kihagi, Erick M. O. Muok, Raissa Sorgho, Ina Danquah

**Affiliations:** 1 Transdisciplinary Research Area “Technology and Innovation for Sustainable Futures” and Center for Development Research (ZEF), Rheinische Friedrich-Wilhelms University of Bonn, Genscherallee 3, Bonn, 53113 Germany; 2 The Faculty of Medicine, University of Bonn, Bonn, Germany; 3 Heidelberg Institute of Global Health (HIGH), Medical Faculty and University Hospital, Heidelberg University, Heidelberg, Germany; 4 Center for Global Health Research (CGHR), Kenya Medical Research Institute (KEMRI), Kisumu, Kenya; 5 Public Health Institute (PHI), Center for Wellness and Nutrition (CWN), Sacramento, CA USA

**Keywords:** Climate change, Agriculture, Home gardening, Perceptions, Qualitative, Rural Kenya

## Abstract

**Background:**

Home gardens are promoted as a strategy for climate change adaptation in SSA. Here, we determined the lived experiences, the perceived knowledge gain and practice change, and the suggested strategies for maintenance, spread and scaling among beneficiaries, implementers and stakeholders of a home gardening intervention in rural Kenya.

**Methods:**

For this explanatory qualitative study, we conducted two focus group discussions (FGDs) with stakeholders (*n* = 5) and implementers (*n* = 8), and 30 in-depth interviews (IDIs) with male (*n* = 5) and female (*n* = 25) beneficiaries living in Siaya county, from September to November 2023. We used purposive sampling and employed semi-structured interview guides. The data were translated into English, transcribed verbatim, and analyzed using inductive content analysis; we mapped the findings along the proposed impact pathways.

**Results:**

The participants articulated good understanding of the trainings and valued them as an occasion for knowledge exchange. The adoption of tailored garden structures, organic gardening, and food preservation created feelings of fulfillment, women empowerment, and family peace. Experienced challenges included water scarcity, ineffective pesticides, and long distances between the beneficiary households. Perceived benefits were increased income, cost savings, and increased dietary diversity. For maintenance, spread and scale, the participants suggested agri-business, local partnerships, and the integration of home gardens into the county’s political agenda.

**Conclusions:**

This project seems to have followed its planned pathways to improved child nutritional status. The organic gardening approach offers solutions in low-resource settings but creates challenges for maintenance, spread and scale of home gardens in Siaya county, Kenya.

**Supplementary Information:**

The online version contains supplementary material available at 10.1186/s41043-026-01263-4.

## Introduction

 Agriculture builds the basis of all food systems. In Kenya, agriculture contributes 26% to the gross domestic product (GDP); it is a source of livelihood to approximately 80% of the population [1]. The majority of farmers practice small-scale rainfed agriculture, lack intensification, and have low adaptative capacities to climate change [1, 2]. The increasing vulnerability of the agricultural sector to climate variability has resulted in low agricultural productivity marked by perennial crop failures, leading to increased food insecurity at the household level [1–4]. In 2014, 18% of the population living in Kenya experienced food insecurity; this has increased to 21% in 2019 [3]. A study from 2021 reports that COVID-19 exacerbated food insecurity in Kenya to 38% [5], while jeopardizing food access and availability, especially for the majority of poor households in Siaya County and other rural areas in Kenya [6, 7]. The result is a high burden of child undernutrition (26%) in the country [8]. According to the 2022 Kenya Demographic and Health Survey (KDHS), 18% of children under five years in Kenya have stunting (chronic undernutrition), 5% have wasting (acute undernutrition), and 10% have underweight (general undernutrition) [9]. In Siaya County, these numbers are 19% for stunting, 2% for wasting, and 7% for underweight [9].

Indeed, climate change impacts child nutritional status in sub-Saharan Africa (SSA) through direct and indirect pathways [10]. This development will likely slow down the reduction of undernutrition in the most vulnerable population groups of young children and women at childbearing age [11]. Thus, identification and implementation of adaptation strategies are imperative to counter the devastating effects of climate change on nutrition and health. According to the Intergovernmental Panel on Climate Change (IPCC), adaptation is defined as “actions and strategies for adjusting to actual or projected climate change-related impacts and their deleterious consequences” [12].

Home gardens or kitchen gardens are one potential adaptation strategy to combat food insecurity in SSA [13, 14]. Home gardens refer to a mixed-cropping system that encompass vegetables, fruits, plantation crops, spices, herbs, ornamental and medicinal plants and sometimes small livestock that can serve as supplementary sources of food and income [15]. According to Mitchelle and Hanstad, the main characteristics of home gardens are: [1] location near the homestead [2], high biodiversity/species richness [3], yields are used for household supplementary consumption and income [4], consisting of a small land area, and [5] representing production systems which can be adopted with little inputs [16]. Owing to their ecological friendliness, low capital intensity and simple technology, home gardens are a promising tool for poor rural households to obtain adequate nutritious food while adapting to the changing climate [15, 17]. In the multi-center, randomized controlled trial “ALIMUS – We are feeding!”, home gardens were implemented in Siaya county, rural Kenya to improve child nutritional status [18]. The home gardening component of the ALIMUS trial involved the establishment and maintenance of small plots (maximum 40 m^2^) near the house to grow fruits and vegetables over a period of 2 years. The project collaborated with the local non-governmental organization (NGO) *Center for African Bio-Entrepreneurship (CABE)* and the Ministry of Agriculture (MoA) of Siaya county. The implementers were responsible for training, issuance of inputs, follow-ups and monthly monitoring of at least 20 beneficiaries assigned to them. Beneficiaries were defined as parents or primary caregivers of under-fives living in the selected villages of Siaya county, while garden leaders denoted the implementers. They were experienced farmers, who trained other members of the community on home gardening.

The intervention involved both, theoretical trainings and practical aspects of gardening. The trainings of the beneficiaries comprised the selection of garden types, botanical pesticide production, seed savings and multiplication, composting, land preparation, weeding and plant protection, harvesting, and preservation. We purposefully used climate-friendly and sustainable inputs for fencing and construction. Through a participatory approach, the beneficiaries and implementers selected indigenous crops meeting their dietary preferences and ecological conditions of their areas. The inputs were sourced from local seed suppliers and producers. Finally, agricultural ward officers from the local MoA worked in close collaboration with the implementers to provide regular technical support to the individual beneficiaries.

The theory of change relies on five proposed impact pathways of the home gardening component comprised (i) inputs: seeds and gardening tools, (ii) processes: horticultural training and supportive supervision, (iii) outputs: knowledge gain, practice change, improved and developed gardens, and increased fruit and vegetable production, (iv) outcomes: increased consumption and income from surplus production, and finally (v) impact: improved child nutritional status [18].

These impact pathways remained to be verified in order to develop strategies for maintenance and scaling. Qualitative research provides a valuable approach to generate in-depth understanding of the proposed impact pathways. Therefore, we conducted an explanatory qualitative study to explore the lived experiences and the perceived impacts of the home gardening intervention among beneficiaries, implementers, and stakeholders in Siaya county, Kenya. The specific objectives were to (1) describe the experiences of home garden implementation; (2) define knowledge gain from the implementation of home gardens; and (3) determine the strategies for maintenance and scaling of home gardens.

## Methods

### Study design and setting

We employed an explanatory qualitative study design to understand the causal mechanisms that support the impact pathways towards improved child nutrition. The study was conducted in Siaya county, located in the south-western part of Kenya and in the larger Lake Victoria basin. According to the latest data from the Kenya National Bureau of Statistics (KNBS), approximately one million people live in Siaya county [19]. Over 90% of the residents are from the Luo ethnic group and 45% are below 14 years. The dominant language is Dholuo, with Swahili and English as the next main spoken languages. Agriculture is the mainstay of the county’s economy, providing employment for most residents. Agricultural activities include crop and livestock production complemented by fishing [19].

**Fig. 1:**
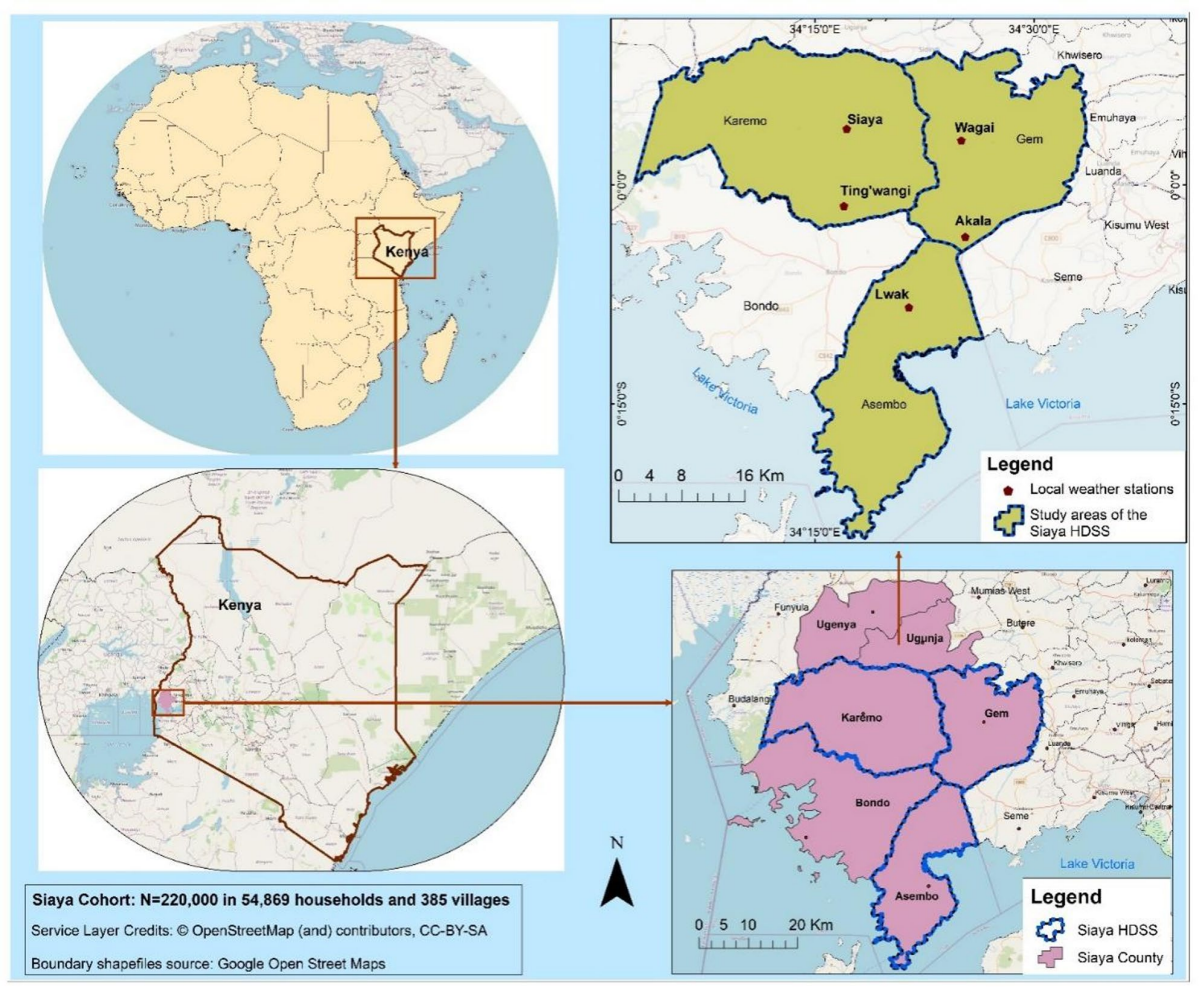
Map of the study area – Five regions (Siaya, Ting’wangi, Wagai, Akala, Lwak) within the area of Siaya Health and Demographic Surveillance System (HDSS)

## Sampling and recruitment

One group of study participants were the beneficiaries of the home garden and nutrition counselling intervention. They were recruited from the five regions of Asembo, Wagai, Ting’ Wang’i, Gem and Karemo within the Siaya Health and Demographic Surveillance System (HDSS) (Fig. 1).

We applied purposive sampling to select beneficiaries for In-depth Interviews (IDIs). Afterwards, we recruited garden leaders and stakeholders for the Focus Group Discussions (FGDs). The eligibility criteria for this qualitative study were (1) co-designing and/or participating in the ALIMUS home gardening component, (2) being conversant in Luo, Swahili or English, being at least 18 years of age, and 4) providing informed written consent. This study was embedded in a cluster-randomized controlled trial (cRCT) on the effects of an integrated agro-biodiversification and nutrition counselling program to improve child nutrition status [18], which recruited participants within a 5-km radius of one of five local weather stations.

## Data collection

We employed semi-structured interview guides, made up of broad topics with key probing questions. The interview guides were piloted prior to application. We collected socio-economic and demographic information of the participants, including age, sex, education, marital status, and occupation. All interviews and discussions were audio-recorded using Philips Audio Recorder (DVT1120) and stored on a password-protected cloud system.

### In-depth interviews:

Two well-trained and linguistically matched, male and female, researchers (EA, GWK) conducted IDIs with the beneficiaries at their homes from September to November 2023. One researcher interviewed the caregiver, and the other one took notes to document the process, key actions, and other important observations. The data collection team held daily debriefing meetings along with memo writing to evaluate the progress, check interview completeness, reflect on the available data and gain perspective on data saturation. In total, we conducted 30 IDIs with male (*n* = 5) and female (*n* = 25) beneficiaries. The mean length of the interviews was 63 min, ranging from 39 to 86 min.

### Focus group discussions:

Two FGDs with implementers (*n* = 8) and stakeholders (*n* = 5) were conducted in English or Swahili in a seminar room at a prominent hotel in Siaya, Siaya County. The first FGD with the stakeholders lasted 120 min while the second FGD with the implementers took 134 min. We did not have any participants who refused to participate, dropped out or requested for an interview rescheduling in this study hence we did not have any repeat interviews.

The researchers established a cordial relationship with respondents prior to the interviews start. Only the respondents of either IDIs or FGDs together with the interviewers were present in the rooms during the interview processes. The two interviewers had interacted with the respondents during the implementation process where they had explained their roles, qualifications, experiences and objectives in the study.

### Consent:

Study information and consent forms were shared prior to the interviews. The study team explained the forms and answered any questions from the participants. For illiterate participants (2 out of 30), a literate witness (family member of the participant) was invited to support the consenting process. Interviewers also informed the participants about the confidentiality of their data and their right to withdraw from the study at any point without consequences. In addition to the written informed consent, all participants consented to audio recording of the IDIs and FGDs.

### Data analysis

The audio recordings were transcribed verbatim and translated from Dholuo and Swahili to English by two local transcribers with each 4 years of translation experience. The final transcripts were stripped off the identifiers and uploaded for analysis to the NVivo software (version 14.23.0). We employed inductive content analysis to uncover the salient concepts of information provided by the interview participants, which are not dictated by pre-existing ideas or concepts. This was a gradual process, which started during the field activities and involved ongoing revision and evaluation. This approach allowed the research team to identify emerging patterns, themes and concepts that complement the proposed impact pathways. First, the interview transcripts, field notes and observations were read, re-read and reviewed. Then, we identified codes (meaning units) using direct content analysis. The codes were applied to the data directly to allow focus on the content of the interview [20]. After coding, we developed summary categories which built into themes. These themes allow concise presentation of relevant subjects and allowing comparison between the participant groups. We finally conducted triangulation between the two coders (EA and GWK) who analysed the data and two co-authors (RS, ID). This was particularly important to remove any potential subjectivity [21, 22].

In June 2024, the lead investigator (EA) conducted a dissemination workshop at a prominent hotel, Siaya Kababa, in Siaya Town, Siaya County. Themes and key findings were presented to the participants for feedback, verification and validation. Participants did not receive their transcripts post interview but were presented with the results of the analysis during the results dissemination workshop. To enhance strict adherence to quality of our reported findings, we applied the 32-item Consolidated Criteria for Reporting Qualitative Research (COREQ) guidelines (Supplementary Material 1) in the entire process of this qualitative research [23]. The checklist is categorized into three domains: (1) research team and reflexivity, (2) study design, and (3) analysis and findings which help researchers focus on relevant areas that must be reported in qualitative research [23]. A page number is indicated on where each item is recorded otherwise ‘not applicable’ (N/A) is indicated in instances where an item is not reported.

## Results

We present our results based on responses and quotes from the study participants (Anderson, 2010). We label our quotes as participant category (beneficiary, garden leader or stakeholder), study specific ID (01–30 for beneficiaries, 01–08 for garden leaders or 01–05 for stakeholders), sex, and age.

### Demographic and socio-economic characteristics

There were 43 study participants: 30 beneficiaries, 8 implementers and 5 stakeholders. Table 1 shows their demographic and socio-economic characteristics. Most of the beneficiaries were mothers and were married. Half of them did not have prior home gardening experience (17 of 30) and attained a primary level of education (14 of 30), respectively. Half of the implementers attained college level of education (4 of 8), all were married, and all had prior home gardening experience. Five of the implementers also served as beneficiaries. The majority of stakeholders were married (4 of 5), all had a college level of education and prior home garden experience (Table 1).

**Table 1:** Socio-demographic characteristics of the study participants

Characteristics	Beneficiaries	Implementers	Stakeholders
N	30	8	5
**Categories**			
	Father (5)	Implementer only (3)	MoA staff (3)
	Mother (21)	Implementer and Beneficiary (5)	Local NGO staff (2)
	Grandmother (4)		
**Women (n)**	25	3	3
Median age (range) in years	36 (27–58)	35 (29–50)	46 (36–59)
**Marital Status**			
Married	25	8	4
Divorced or widowed	5	0	0
Unmarried	0	0	1
**Educational Level**			
Primary	14	1	0
Secondary	12	3	0
College/University	2	4	5
**Prior home gardening experience (yes)**	13	8	5

Numbers indicate count data

MoA, Ministry of Agriculture; NGO, non-governmental organization

### Experiences of home garden implementation

The participants had varied experiences during the intervention. Overall, participants expressed interest in the home gardening intervention. The trainings were useful in acquiring new information, improving knowledge and contributing to new agricultural practices. Moreover, participants derived several benefits as a result of participating in the intervention. In spite of these, participants also enumerated on the challenges they experienced during that period.

#### Easy topics and valuable launch point – perceptions of training sessions

The study participants expressed interest in all of the offered training topics. Beneficiaries found the training topics simple to understand and to practice, because the raw material for the trainings in their home gardens were easily available, “*making organic pesticides was my favorite topic because it was simple. The requirements (input materials) are available.” (Beneficiary_012_F_44).* Stakeholders shared that the trainings were an important launch point for them to expand their knowledge and explore locally available resources “*On crop protection*,* the use of locally available plants like Tephrosia to come up with a bio-pesticide. This was a good topic because from that*,* I have done more research*,* I have more linkages to get more plants which our farmers can use*,* locally available*,* to protect their crops against pests and diseases. So*,* this was the most valuable lesson to me” (Stakeholder_005_M_46).*

##### Water, crops and space – novel practices for home gardens

Beneficiaries shared the changes they have implemented, centering around the expansion of their agricultural skills such as mulching and making seed beds, *“I learnt that I could do mulching on the vegetables. No matter how dry the weather is*,* the wastes that I put in retain some water. So*,* I was planting vegetables even during the dry season*,* and they remain wet” (Beneficiary_027_F_31).*

Implementers emphasized that garden variety and crop variety have increased, “*initially some households were just growing one type of vegetable but by the time we joined and trained them*,* they started growing different types of vegetables. Today you eat this one*,* the next day you eat that one then it continued in that manner. Those vegetables had a lot of nutrients and the malnourished state went down because they are eating different types of vegetables not only eating cowpeas all day throughout the week. Right now*,* they are eating different types of vegetables” (Garden Leader_002_M_42).*

Stakeholders leveraged their improved knowledge to promote new foci in gardening, such as space saving structures and promoting organic approaches, “*I’m really promoting vertical gardens and cone garden and so many people are into it. And through this project*,* it has changed my way of operating. It is purely use of organic products to protect those vegetables against pests and diseases. And even its nutrition*,* it is purely organic” (Stakeholder_005_M_46).*

##### Fulfillment, independence and empowerment – perceived benefits

Implementation of home gardens led to perceived nutritional, economic and social benefits to participants, which are summarized in Table 2. In terms of the nutritional benefits, the interviewees reported enhanced dietary diversity, making their meals more complete and satisfying. The participants also reported increased access to vegetables, relating to both, physical access (near the home) and financial affordability (self-produce). Regarding nutritional benefits, the interviewees felt that vegetables from their home gardens were safer and thus, more nutritious for their families. For the economic benefits, the study participants talked about increased incomes from the sale of surplus vegetables. Some beneficiaries even articulated to consider starting a vegetable business. Participants also reported financial savings, because they had to spend less for the purchase of vegetables on the market. According to the participants, the home garden intervention had the following social benefits. The project created feelings of emotional fulfillment in owning a home garden and producing vegetables. Especially among the female participants the home gardens provided an increased sense of self-reliance and independence. Producing one’s own vegetables was essential in providing feelings of security and safety for girls and young women, because they did not have to go far from their homes to harvest vegetables from the fields. The female participants shared that the intervention enhanced peace among couples, because they did not have to completely rely on their husbands for monetary needs due to income from the garden. If they had to ask for money from their partners, this could lead to misunderstandings, which was mitigated through the home garden income. The sense of self-reliance and women empowerment became a motivator for continued effort to sustain the home gardens.

**Table 2:** Perceived benefits of home gardens

Category of benefits	Codes from transcripts	Selected quotes from respondents
Nutrition	Dietary diversity	*“They [home gardens] have helped me get a meal*,* we were also allowed to sell when they were in surplus so that you buy a different meal and change your diet. You can buy eggs or even fish with the money you have gotten from the vegetables” (Beneficiary_025_M_31)*
	Access to vegetable	*“At the time you don’t have the financial resources*,* or you come back home late*,* instead of sleeping hungry*,* you will be able to get vegetables because it’s within your reach no matter the time” (Beneficiary_018_F_36)*
	Nutritious vegetables	*“It is purely use of organic products to protect those vegetables against pests and diseases. And even its nutrition*,* it is purely organic and they (vegetables) are very nutritious” (Stakeholder_005_M_46).* *“I could teach some of them on how to plant vegetables and what they needed to do so that they can also help other children to get the right nutrients” (Garden Leader_003_M_33).*
Economic	Income generation	“*I am selling vegetable to farmers*,* and they have always helped me so I don’t spend much. I am the one getting money from other people and I realized that through vegetable multiplication I can make money from it and it’s a job on its own” (Garden Leader_004_ M_33)*
	Costs saving	“*…it [my home garden] has reduced the cost of buying vegetables and frequent asking of money [by the spouse] when am leaving the house in the morning” (Garden Leader_007_M_37)*
Social	Emotional fulfilment	*“It feels good knowing that you have vegetables*,* you can always pluck any time” (Beneficiary_011_F_31)*
	Risk reduction	*“Once I plant it*,* I know that it can help*,* because sometimes I don’t have money and my other child has to go look for vegetables in the bush. Something (like a snake) might bite her in the bush” (Beneficiary_007_M_45).*
	Women empowerment	*“Right now*,* I plant my vegetables*,* eat some and sell some. The money I generate from there helps me to take care of some of the things needed in the house rather than asking my husband for it. It has become of great help to me and I really thank the project for the new things it has taught me” (Garden Leader_006_F_50)*
	Community enhancement	“*…if one is empowered through the work you are doing of home gardening and gets to set up their own*,* it reduces cases of theft and reduces on spending on vegetables.” (Garden Leader_001_M_29)*
	Peace among couples	*“Home garden is a peace maker. It has empowered women to get money from selling part of the produce and enabling them to have something of their own not all the time asking the husband for money” (Garden Leader_001_M_29)*

##### Water scarcity, ineffective pesticides, physical distance – experienced challenges

Participants enumerated some challenges they encountered during the implementation phase: water scarcity, physical distance, ineffective pesticides, and the political situation at the time as show in Table 3. Indeed, in northern areas of the study area, access to water was more challenging than in the southern parts. Some participants noted that the botanical pesticides were not fully effective against all pests. Garden leaders and stakeholders particularly noted that the study area was quite vast. The wide area was especially a challenge during the group trainings and equipment distributions, because some households had to travel long distances with their children to attend the trainings or pick up the equipment (Table 3).

**Table 3:** Experienced challenges of home garden implementation

Coded challenge	Selected quotes from respondents
Climate variability	“*The difference in the geological area*,* sometimes you find some areas are very dry that even doing irrigation sometimes becomes very hard” (Stakeholder_001_ M_29)*
Limited effects of botanical pesticides	*“Many times*,* you may get a pest invading a vegetable from the midrib or through the leaves because the botanical ones are only working on the upper part but not inside*,* if you get a pest invading the inside of that vegetable*,* that vegetable will end up drying up” (Garden Leader_001_M_29)*
Socio-political issues	*“The equipment distribution started during the campaign period and most of the politicians were using it as a campaign tool claiming that they were the ones distributing it which made some people feel bad because they were being forced to vote for the individuals who they claimed were distributing them which was not the case” (Stakeholder_006_F_50)*
Wide geographic area of implementation	“*The distances are so wide*,* so reaching out to these farming households may not be easy. Like when you bring them together for training*,* you try following them up*,* it makes you so tired to reach out to each one of them” (Stakeholder_003_F_59)*

### Knowledge gain

Knowledge exchange has been a critical component during the implementation of home gardens. The training sessions with the participants offered an opportunity to gain new insights from the trainers but also it fostered learnings among the participants themselves. The participants enumerated that as they were divided into smaller clusters, they were able to cross-check and reference with each other regarding the training sessions. Additionally, the practical sessions on the gardens equipped beneficiaries and garden leaders with hands-on skills which were all necessary as they went ahead to implement their own gardens. These sessions were also important as they enhanced the replicability of the learnings hence making it easier for them to implement on their own. Finally, the participants expressed that they have been able to transfer the knowledge gained into their own work.

#### Contextualization and organic solutions – perceived knowledge gain

Beneficiaries and garden leaders defined new knowledge as new learnings, meaning information they had never heard of, or known prior to the intervention. For the stakeholders, it represented a change in perspective, a shift from the conventional viewpoint to a more localized approach in resolving challenges.

Beneficiaries learned to use and optimize locally available resources to construct home gardens and to prepare organic manure *“We were told how to prepare the garden and how to grow vegetables in the seedbed. This one I didn’t know*,* so I left there with a lot of knowledge. We were taught how to prepare manure*,* we no longer use artificial fertilizer*,* I prepare manure here and used it to plant vegetables and maize*,* the maize did so well*,*” (Beneficiary_030_F_30).*

Garden leaders learned of new home garden models, set ups and structures which are locally practicable, *“before the training*,* I thought that for one to practice kitchen garden*,* you are required to have a large chunk of land not knowing that that isn’t the case. Kitchen garden could also be practiced in a bag” (Garden Leader_004_M_33).* A beneficiary also alluded that the new models of home gardens were easy to maintain, *“I did not know that vegetables can be grown in bags… because with the sacks*,* even if there is drought one can use less water and the plants survive” (Beneficiary_020_M_40).*

Knowledge acquisition for garden leaders also occurred around organic practices such as composting, “*the new thing I learnt is vermicompost where we were decomposing. This was a method to produce manure through the white worms which is the earth worms” (Garden Leader_003_M_33)* and pests’ control. *“Before the training*,* I only knew that pests could be controlled by the use of manufactured pesticides bought from agrovet. Then when we attended the training*,* we were taught something called the botanical which includes the processes to follow when making the home-made pesticides. This was something very new to me and even the processes were quite unique” (Garden Leader_004_M_33).*

Stakeholders were not mandated to participate in the garden trainings. Some attended and shared that they learnt new practices such as preservation, *“In my entire participation in this project*,* I learnt a lot of things like I came to learn a lot in value addition trainings” (Stakeholder_001_M_46).*

#### Me, my family, and neighbors – application of knowledge gain

A beneficiary noted that as a teacher, she is using the knowledge acquired during the intervention to train her students especially those taking agriculture courses, *“I have used this information in school. I will always talk more about school because I want my learners also to have experience. So*,* upper-class learners do agriculture*,* so when they want to make the seedbed the agriculture teacher connects the ideas” (Beneficiary_023_F_31).*

Beneficiaries are also applying their new knowhow to better manage their gardens and train other community members, *“This new information has helped me [improve] on how I can manage my home garden and… I have been able to share it with people from my surrounding when they come to consult because it is not working for them yet. When they look at what am doing*,* its yielding fruits” (Beneficiary_010_F_41).*

As a result of the gained knowledge, beneficiaries have increased vegetable consumption in their households and this has led to perceived improvement in the nutrition and health of their children, “*I thought that a child should only eat other things apart from vegetables*,* but I discovered that her eating the vegetables had boosted her immune system to the level that she doesn’t get sick regularly” (Beneficiary_013_F_38)*.

Stakeholders noted that they have leveraged their intervention knowledge to advocate for policy considerations towards volunteer community garden leaders, “*it’s a high time that we also advocate for these volunteer peer trainers among farmers so that they can also be supported” (Stakeholder_004_F_54).*

### Maintenance, spread and scaling

In pursuit of long-lasting benefits of the home gardens, study participants enumerated on mechanisms they would employ to maintain, spread or upscale the interventions within their area and beyond. These strategies would ensure that the overall benefits of the home gardens last beyond the involvement of the external support and partners.

#### Garden business, local partnerships and policy integration – strategies for maintenance, spread and scaling

The strategies highlighted by study participants to sustain home gardens are in two levels. The first level refers to the internal or household-level strategies that the participants can do on their own. The second level comprised external, structural, institutional and material support that they would need in order to sustain the home gardens as indicated in Table 4. At the household-level, beneficiaries and garden leaders explained that they could leverage on the knowledge they have acquired, they would practice seed bulking and seed saving techniques, and commercialize and form enterprises to be able to sustain the home gardens.

In terms of the external or structural support, study participants enumerated that they would need to be linked to the local county governments. There is need for creating awareness through mass media or public forums and building synergies between agro-nutrition partners within the county.

Finally, the participants emphasized that it is important to anchor home gardening within the county agricultural sector development plans to ensure sustained home gardening in the county. All these strategies weigh in towards ensuring continuity of the home gardening beyond the involvement of external partners and fostering self-reliance (Table 4).

**Table 4:** Suggested strategies for maintenance, spread and scaling of home gardens

Level of activity	Strategy	Selected quotes from respondents
Household	Seed saving and seed bulking	“*Something like crotalaria has seeds when it grows. I always preserve and I have some which I can plant even when others delay. Something like cowpeas*,* it also creates seeds. So*,* you find that they are readily available and I just plant them. There’s no single time that I lack vegetables.” (Beneficiary_005_F_30)*
	Leveraging on acquired knowledge	*“We have already been taught how to prepare a kitchen garden*,* how to take care of children. In the next generation*,* we just continue with what we were taught… the teachings have remained with us. So*,* we just continue practicing. We can also teach other people who were not in the program” (Beneficiary_005_F_30)*
	Commercialization and development of enterprises	“…*If it could be increased*,* in the quantity and the acreage so that these people are able to know that you need to produce for commercial*,* get a little more money so that you buy other things that you need in the household” (Stakeholder_003_F_59)*
Institutional	Linkage to local county government structures	*“Since agricultural sector is devolved*,* the organization [referring to the researcher’s organization] can link us to the county government being that we have trained well. We can be used as trainers to train other people so that they also have food” (Garden Leader_006_ F_50)*
	Building synergies between partners within the agro-nutrition space	“*We have several partners in the Agri-nutrition space and also promoting kitchen gardening. We can bring these partners together. Let them form a consortium or something where they are sharing feedback*,* sharing ideas so that it is a structure that will continue” (Stakeholder_004_F_54)*
	Mass education and public forums	*“You can have a radio talk show to discuss these issues teaching women to be aware of what is going on.” (Beneficiary_008_F_41); “I can also use it in the church*,* for instance*,* the church that I go to*,* we have youths there*,* and am one of them*,* I can also teach them there”. (Beneficiary_017_F-29)*
	Anchoring home gardens on the county development plans	“*Including this home gardening aspect in our county plans is one aspect of sustainability” (Stakeholder_004_F_54)*

#### No staff, no money, no inputs – challenges to maintenance, spread and scaling

The participants elaborated on the challenges that they would experience or have already experienced in their quest to sustain the home gardening practice. Some of the challenges included:

- **Inaccessibility of quality seeds**: stakeholders noted that beneficiaries could have a challenge to access quality seeds to ensure continued home gardening, *“And the second biggest challenge in sustainability is access to quality seeds and clean planting materials” (Stakeholder_002_F_43).*
- **Water scarcity**: stakeholders explained that there is severe drought in some parts of the region leading to great water scarcity, *we have to think about technologies around water or what kinds of models of home gardens we can adopt that align to the reality that water is a very scarce resource in the county” (Stakeholder_002_F_43).*
- **Limited technical support and inputs from county government**: garden leaders noted that the county government will not offer them the much-needed support so that they can continue with the home garden activities, “*if you hand us over to the county government*,* I highly doubt it if county government will provide us with the required seeds needed for all these households. Secondly*,* I doubt it if we are going to be able to attend these trainings as you used to do to us” (Garden Leader_007_M_37).*
- **Inadequate staff at county level to support maintenance initiatives**: stakeholders indicated that there are not adequate staff at the county to support all the sustainability initiatives, *“the first challenge I see is that we don’t have enough extension officers that will be able to disseminate this information to the larger 300*,*000 farming households” (Stakeholder_004_F_54).*
- **Financial constraints**: stakeholders alluded that the county is facing financial constraints and this could limit their capacity to offer support to beneficiaries, “…*allocating funds towards home gardens can be a challenge so you have to keep reaching out to partners and because of also differing priority in the county*,* you may not get the amount of facilitation you are envisaging to have an upscaled program” (Stakeholder_004_F_54).*

## Discussion

### Summary of main findings

Home gardens are promoted as a strategy for climate change adaptation in SSA. Within a home garden intervention in Siaya county, Kenya, we determined the lived experiences, the perceived knowledge gain and practice change, and the suggested strategies for maintenance and spread among beneficiaries, implementers and stakeholders. Trainings on home gardens were easily understood and were valued as an occasion for knowledge exchange. Beneficiaries, implementers and stakeholders were enthusiastic about the perceived benefits of increased income, cost savings, and enhanced dietary diversity of the family. Despite the challenges of water scarcity, ineffective pesticides and large distances between the beneficiary households, the familiarization with new horticultural techniques and crops created feelings of fulfillment, independence, and empowerment. Perceived knowledge gain covered the contextualization of gardens and organic solutions for fencing, fertilization, pest control, and preservation. Beside the limitations in this resource-poor context, it appeared realistic to maintain, spread or scale the home garden concept through establishing garden business, fostering local partnerships, and integrating home gardens in to the county’s political agenda.

### Impact pathways of home gardening for improved child nutrition

**Fig. 2:**
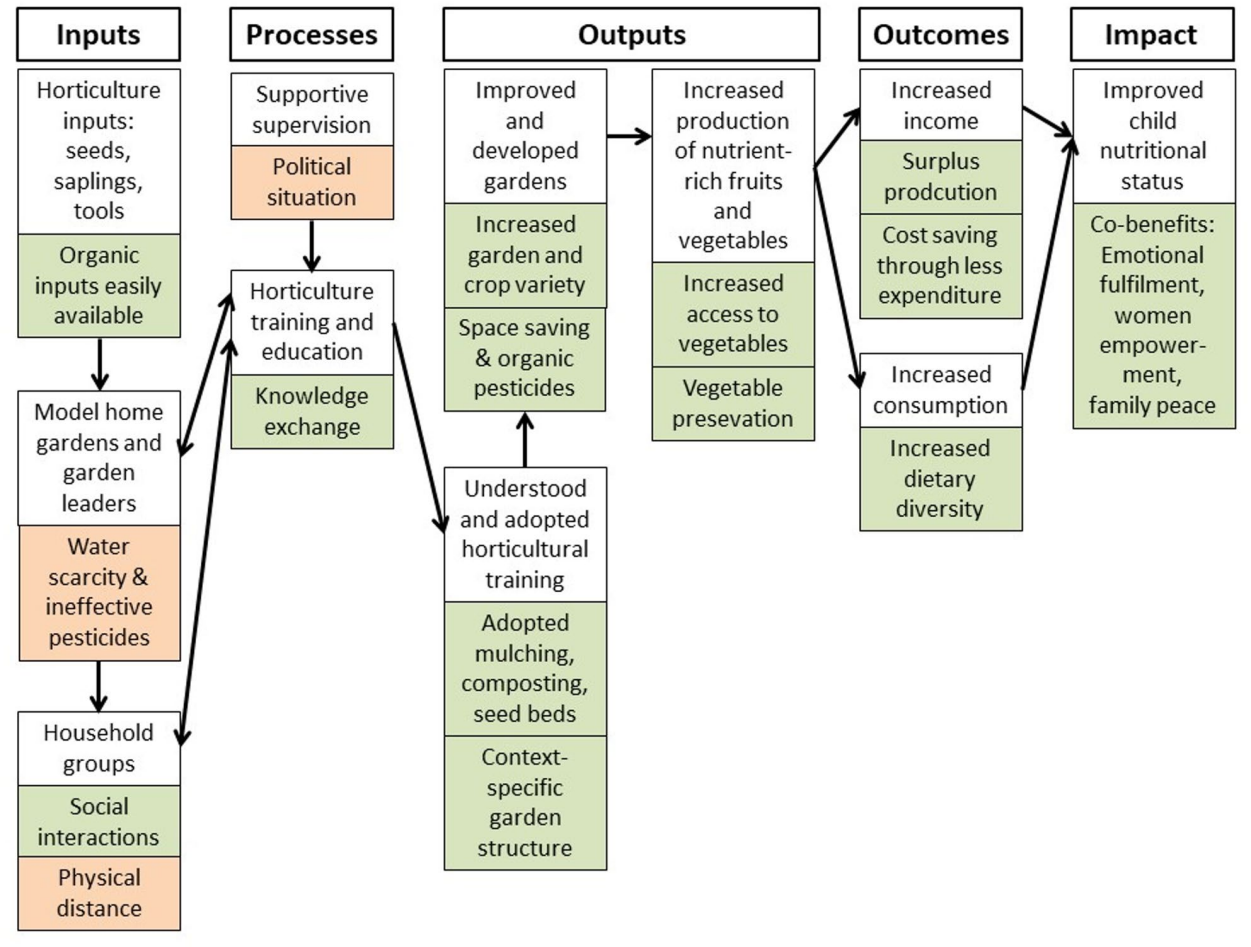
Impact pathways of home gardening for improved child nutrition among beneficiaries, implementers, and stakeholders in Siaya county, Kenya. **white boxes**: steps in the impact pathway; **green boxes**: positive perceptions by the participants; **orange boxes**: negative perceptions by the participants.

Our findings about lived experiences, knowledge gain, and practice change among beneficiaries, implementers and stakeholders provide in-depth understanding of the impact pathways of this home gardening intervention in Siaya county, Kenya. Figure 2 provides the graphical summary of the important steps in the perceived pathways to improved child nutritional status.

#### Inputs, processes, outputs and perceived challenges

The environmentally friendly inputs and processes strongly contributed to the perceived benefits of this home gardening intervention. Horticultural inputs were organic seeds, organic fertilizer, and organic pesticides; infrastructural inputs included natural fencing material amongst others. Indeed, organic agriculture is on the rise in LMICs and constitutes an important element of climate-friendly adaptation strategies [24, 25]. In this study, model gardens and household groups provided opportunities for social interaction, which constitutes a proven success factor for home gardens [26]. The lack of natural, financial and logistical resources were major drawbacks for the interviewees. Indeed, in the same study area, water scarcity has been identified as a potential barrier to home gardening interventions [27]. The importance of regular exchange and closeness between beneficiaries and implementers [28] is mirrored by the complaints about the physical distances between the intervention households in our study. In addition, local partnerships, training and education immensely determine the impacts of home garden interventions in SSA [27, 29, 30]. Yet, for the participants in this study, long-term perspectives for supportive supervision were questionable due to the political transition between two legislative periods. Political goodwill and policy support have been found to be paramount for longevity and sustainability of home gardens throughout SSA, including Benin and Uganda [24, 31], Ethiopia [32], and South Africa [33]. This is also supported by synthesized evidence on approaches to enhance home gardening in Africa, Asia and Latin America [15].

The understanding and adoption of horticultural training were key in the mapped impact pathways of the ALIMUS study [18]. The participants revealed that mulching, composting, and the establishment of seed beds were important adopted practices. Further, the tailoring of garden constructions to the specifics of the compound were perceived as an important learning lesson. These findings are corroborated by evidence from Ghana and other parts of Kenya, emphasizing the importance of education, training, and practical experience for the adoption of new practices [30, 34]. As a result, the interview participants in this study experienced improved and developed gardens. The respective features were diversified types of gardens, increased vegetable variety, space saving, and use of organic pesticides. This speaks again for the popularity of organic agriculture in SSA [24, 25], and widens the lens to urban areas, where the efficient use of space is even more important [35]. Consequently, our study participants reported increased production of food crops, as demonstrated by enhanced physical access to vegetables, and – as a result of food preservation methods – increased stability of this access. Although many projects have reported similar gains for their participants, some households struggle to establish and maintain home gardens. A qualitative study in South Africa revealed that cultural constraints may contribute to the failure of home gardens. People perceived large plots only to be suitable for food production; they considered gardens as a means of decoration, and heavily relied on food purchasing [33]. A systematic review on projects in developing countries reported mixed results on the effectiveness of home gardens in urban environments to enhance food security, dietary diversity and nutritional status [36]. The study confirms positive association between urban farming, dietary diversity and food consumption. Yet, the findings for nutritional status were mixed [36], emphasizing the importance to triangulate our qualitative data with quantitative measures of effectiveness.

### Perceived benefits, outcomes and impact

The immediate outcomes of home garden interventions have been seen in other gardening projects in SSA, such as increased income through surplus production of foods [37]. Also, cost savings due to less food expenditure have been reported from gardening projects in LMICs [29, 30, 38]. In the present study, enhanced dietary diversity was articulated and proves increased vegetable consumption. For the vulnerable target group of young children, who are particularly threatened by the impacts of climate change on nutrition [11], dietary diversity supports the adequate intake of critical nutrients. As demonstrated in Bangladesh, Nigeria, and other parts of East Africa [29, 35, 39], home gardens, indeed, confer beneficial impacts on the nutritional status of young children in subsistence farming families. Importantly, the participants in the present study also reported co-benefits alongside improved child nutritional status. These ranged from emotional fulfilment through women empowerment up to family peace. Empowerment of women has previously been reported from home garden projects in Bangladesh [40, 41] and East Africa [29].

The negative and positive perceptions of this home gardening intervention among our interviewees hint towards the suggested actions for maintenance, spread and scaling of this specific home gardening program in Siaya county, Kenya.

### Strategies for maintenance, spread, and scaling of home gardens

The study participants highlighted several strategies to sustain home gardens beyond the project lifetime. These strategies included linkage to the local county government, integrating home gardening into the county development plans, seed saving initiatives, leveraging on acquired knowledge by the project participants, development of commercial enterprises, mass education, and building synergies between partners in the agri-nutrition space.

These results reflect an increasing recognition of local and community-driven practices for home gardens [15, 42, 43]. It is imperative to empower local leaders and foster community ownership to ensure long-lasting home gardening initiatives. The findings underpin the importance of providing continued and adjusted support to beneficiaries of home garden projects after the initial training phase, especially in areas that are prone to environmental stress [38, 44, 45]. Previous reports from South Africa [46], Uganda [24], Kenya [27], and Ethiopia [13, 32] have highlighted the following challenges to sustain home gardens in SSA – all of which apply to the present context in Siaya county: climatic variability, lack of political will, limited financial and human resources, and inaccessibility of high-quality seeds. Therefore, for sustained home gardens, their spread and scaling, the actors at all levels of the agri-food system should follow the United Nations (UN) principles of sustainable development. These advocate for multi- and cross-sectoral collaboration at all administrative levels, while integrating local agricultural knowledge and practice into the broader policy frameworks [43, 47]. The findings highlight the need for progressive policies that promote the integration of home gardening into broader agricultural and nutritional strategies at the county level. By anchoring home gardens into the local development plans and building strong partnerships among stakeholders, there is potential to scale up these efforts and achieve long-term food security and nutrition goals.

### Strengths and limitations

The findings of this study need to be interpreted with caution. First, the study is explanatory in nature, and thus, the transferability of the findings to other contexts needs to be verified. Second, we conducted most interviews in the Dholuo and Swahili languages for facilitating discussions on culture-specific and sensitive issues. During the subsequent transcription process and translation into English, we may have lost some meanings. To address this limitation, we validated the data through member-checking, debriefings and the employment of local transcribers with several years of experience in data transcription. Another strength of this study is the strict adherence to the established COREQ guidelines during the entire research process, which enhances the transparency and transferability of our findings. Other strengths of this study include robust data analysis through peer coding to reduce individual subjectivity, and the use of two culturally-matched, male and female interviewers for enhanced mining of the interview information.

## Conclusion

This qualitative study provides insights into the specific impact pathways that were experienced by the beneficiaries, implementers and stakeholders of a home gardening intervention in Siaya county, Kenya. The project seems to have followed its planned pathways to improved child nutritional status. These act through gaining and sharing knowledge in horticulture; adoption of new gardening techniques; planting of a variety of vegetables; preservation of foods; increased income; feelings of fulfilment, empowerment and peace; and ultimately, enhanced dietary diversity. The organic gardening approach is appreciated as a low-input strategy. However, the same approach creates challenges for sustaining, spreading and scaling the intervention, including irrigation, effectiveness of pesticides, and quality of seeds. By addressing the challenges identified and building on the perceived benefits of the intervention, there is a great potential for home gardens to act as a climate change adaptation strategy for improved child nutritional status in Kenya and beyond.

## Supplementary Information

Supplementary Material 1

## Data Availability

The data that support the findings of this study are available from the Steering Committee of the DFG-funded Research Unit “Climate change and health in sub-Saharan Africa”, coordinated by Ina Danquah: ina.danquah@uni-bonn.de but restrictions apply to the availability of these data, which were used under license for the current study, and so are not publicly available. Data are however available from the authors upon reasonable request and with permission of the Steering Committee of the DFG-funded Research Unit.

## Abbreviations

CABE: Center for African Bio-Entrepreneurship
COREQ: Consolidated criteria for reporting qualitative research
FAO: Food and agriculture organization of the united nations
FGD: Focused group discussion
GDP: Gross domestic product
HDSS: Health and demographic surveillance system
HIGH: Heidelberg institute of global health
IDI: In-depth interview
IPCC: Intergovernmental panel on climate change
KNBS: Kenya national bureau of statistics
KEMRI: Kenya medical research institute
LMIC: Low-and middle-income countries
MoA: Ministry of agriculture
NGO: Non-governmental organization
SERU: Scientific and Ethics Review Unit
SSA: sub-Sahara Africa
UN: United Nations
WHO: World health organization
WMA: World medical association
